# A new copper(II) complex based on 1-[(1*H*-benzotriazol-1-yl)meth­yl]-1*H*-1,2,4-triazole

**DOI:** 10.1107/S1600536811013511

**Published:** 2011-04-16

**Authors:** Huai-xia Yang, Jun Zhang, Dong Zhao

**Affiliations:** a Pharmacy College, Henan University of Traditional Chinese Medicine, Zhengzhou 450008, People’s Republic of China; b Department of Chemistry, Zhengzhou University, Zhengzhou 450052, People’s Republic of China

## Abstract

The title complex, tetra­aqua­{1-[(1*H*-benzotriazol-1-yl)meth­yl]-1*H*-1,2,4-triazole-κ*N* ^4^}(sulfato-κ*O*)copper(II) sesquihydrate, [Cu(SO_4_)(C_9_H_8_N_6_)(H_2_O)_4_]·1.5H_2_O, is composed of one copper atom, one 1-[(2*H*-benzotriazol-1-yl)meth­yl]-1-*H*-1,2,4-triazole (bmt) ligand, one sulfate ligand, four coordin­ated water mol­ecules and one and a half uncoordinated water mol­ecules. The Cu^II^ atom is six-coordinated by one N atom from a bmt ligand and five O atoms from the monodentate sulfate ligand and four water mol­ecules in a distorted octa­hedral geometry. In the crystal, adjacent mol­ecules are linked through O—H⋯O and O—H⋯N hydrogen bonds involving the sulfate anion and the coordin­ated and uncoordinated water mol­ecules into a three-dimensional network.

## Related literature

For background to complexes based on triazole and benzotriazole derivatives, see: Aromia *et al.* (2011 ▶); Meng *et al.* (2009 ▶). For background to complexes with Cu^II^ atoms, see: Zhou *et al.* (2007 ▶); Brown *et al.* (2009 ▶).
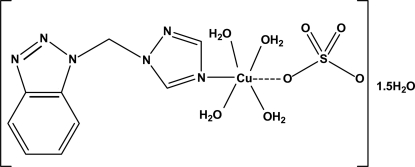


## Experimental

### 

#### Crystal data

[Cu(SO_4_)(C_9_H_8_N_6_)(H_2_O)_4_]·1.5H_2_O
*M* *_r_* = 458.90
Monoclinic, 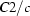

*a* = 12.496 (3) Å
*b* = 8.662 (2) Å
*c* = 31.543 (6) Å
β = 90.97 (3)°
*V* = 3413.7 (12) Å^3^
*Z* = 8
Mo *K*α radiation
μ = 1.47 mm^−1^
*T* = 293 K
0.20 × 0.16 × 0.15 mm

#### Data collection

Rigaku Saturn diffractometer
Absorption correction: multi-scan (*CrystalClear*, Rigaku/MSC, 2006 ▶) *T*_min_ = 0.758, *T*_max_ = 0.810
13448 measured reflections
4046 independent reflections
3502 reflections with *I* > 2σ(*I*)
*R*_int_ = 0.035

#### Refinement

*R*[*F* ^2^ > 2σ(*F* ^2^)] = 0.041
*wR*(*F* ^2^) = 0.088
*S* = 1.10
4046 reflections
240 parameters
H-atom parameters constrained
Δρ_max_ = 0.33 e Å^−3^
Δρ_min_ = −0.45 e Å^−3^

### 

Data collection: *CrystalClear* (Rigaku/MSC, 2006 ▶); cell refinement: *CrystalClear*; data reduction: *CrystalClear*; program(s) used to solve structure: *SHELXS97* (Sheldrick, 2008) ▶; program(s) used to refine structure: *SHELXL97* (Sheldrick, 2008) ▶; molecular graphics: *SHELXTL* (Sheldrick, 2008) ▶; software used to prepare material for publication: *SHELXTL* ▶.

## Supplementary Material

Crystal structure: contains datablocks global, I. DOI: 10.1107/S1600536811013511/bv2183sup1.cif

Structure factors: contains datablocks I. DOI: 10.1107/S1600536811013511/bv2183Isup2.hkl

Additional supplementary materials:  crystallographic information; 3D view; checkCIF report

## Figures and Tables

**Table 1:** Hydrogen-bond geometry (Å, °)

*D*—H⋯*A*	*D*—H	H⋯*A*	*D*⋯*A*	*D*—H⋯*A*
O1—H1*W*⋯O9	0.85	1.82	2.662 (3)	170
O2—H3*W*⋯O10^i^	0.85	1.89	2.723 (2)	165
O9—H9*W*⋯N6^ii^	0.85	2.00	2.836 (3)	168
O10—H11*W*⋯O6^iii^	0.85	1.93	2.771 (2)	170
O1—H2*W*⋯N2^iii^	0.85	2.16	2.951 (3)	155
O3—H5*W*⋯O8^iv^	0.85	1.99	2.789 (3)	156
O4—H7*W*⋯O6^v^	0.85	1.86	2.697 (2)	170
O9—H10*W*⋯O7^v^	0.85	1.99	2.824 (3)	165
O3—H6*W*⋯O6^v^	0.85	2.19	2.943 (3)	148
O2—H4*W*⋯O5^vi^	0.85	1.92	2.766 (2)	174
O4—H8*W*⋯O8^vi^	0.85	1.85	2.702 (2)	175

Symmetry codes: (i) 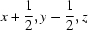; (ii) 
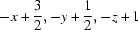
; (iii) 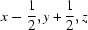; (iv) 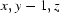; (v) 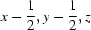; (vi) 
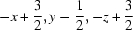
.

**Fig. 1.**
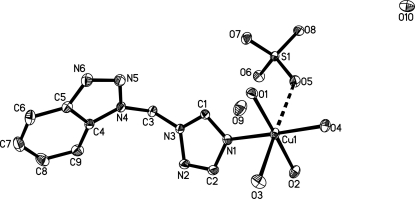
View of the title complex, showing the labeling of the 30% probability ellipsoids. H atoms are omitted for clarity.

## supplementary crystallographic information

### Comment

Triazole and benzotriazole derivatives have been widely used in the construction of complexes since they can act as polydentate ligands and function as bridging ligands (Aromia *et al.*, 2011; Meng *et al.*, 2009). Moreover, Cu^II^ complexes have attracted more and more attention owing to their intrinsic esthetic appeal and potential applications in various fields (Zhou *et al.*, 2007; Brown *et al.*, 2009). In this work, through the reaction of 1-((benzotriazol-1-yl)methyl)-1-*H*-1,2,4-triazole (bmt) with copper sulfate at room temperature, we obtained the title complex [Cu(bmt) (SO_4_) (H_2_O)_4_] (H_2_O)_1.5_, which is reported here. As shown in Figure 1, each Cu^II^ ion is located in a slightly distorted octahedral environment and is coordinated to one nitrogen atom from the bmt ligand, five oxygen atoms from four water molecules and one monodentate sulfate. Atoms O1, O2, O4, N1 and Cu1 are nearly co-planar (the mean deviation from the plane is 0.0203 Å). The apical Cu1—O3 and Cu1—O5 bond lengths (2.331 (2) and 2.465 Å) are considerably longer than the equatorial ones (1.974 (2)- 2.002 (2) Å) due to the Jahn-Teller effect. Intramolecular O—H···O hydrogen bonds stabilize the molecular configuration and O—H···O, O—H···N hydrogen bonds between adjacent molecules consolidate the crystal packing.

### Experimental

The ligand 1-((benzotriazol-1-yl)methyl)-1-*H*-1,2,4-triazole (0.1 mmol) in methanol (4 ml) was added dropwise to an aqueous solution (2 ml) of copper sulfate (0.1 mmol). The resulting solution was allowed to stand at room temperature. After three weeks blue crystals with good quality were obtained from the filtrate and dried in air.

### Refinement

H atoms are positioned geometrically and refined as riding atoms, with C-H = 0.93 (aromatic) and 0.97 (CH_2_) Å and O-H = 0.85 Å, and with U_iso_(H) = 1.2 U_eq_(C,O).

### Crystal data

**Table:**

[Cu(SO_4_)(C_9_H_8_N_6_)(H_2_O)_4_]·1.5H_2_O	*F*(000) = 1888
*M**_r_* = 458.90	*D*_x_ = 1.786 Mg m^−^^3^
Monoclinic, *C*2/*c*	Mo *K*α radiation, λ = 0.71073 Å
Hall symbol: -C 2yc	Cell parameters from 4587 reflections
*a* = 12.496 (3) Å	θ = 2.6–27.9°
*b* = 8.662 (2) Å	µ = 1.47 mm^−^^1^
*c* = 31.543 (6) Å	*T* = 293 K
β = 90.97 (3)°	Prism, blue
*V* = 3413.7 (12) Å^3^	0.20 × 0.16 × 0.15 mm
*Z* = 8	

### Data collection

**Table:**

Rigaku Saturn diffractometer	4046 independent reflections
Radiation source: fine-focus sealed tube	3502 reflections with *I* > 2σ(*I*)
graphite	*R*_int_ = 0.035
Detector resolution: 28.5714 pixels mm^-1^	θ_max_ = 27.9°, θ_min_ = 2.6°
ω scans	*h* = −16→16
Absorption correction: multi-scan (*CrystalClear*, Rigaku/MSC, 2006)	*k* = −8→11
*T*_min_ = 0.758, *T*_max_ = 0.810	*l* = −41→36
13448 measured reflections	

### Refinement

**Table:**

Refinement on *F*^2^	Primary atom site location: structure-invariant direct methods
Least-squares matrix: full	Secondary atom site location: difference Fourier map
*R*[*F*^2^ > 2σ(*F*^2^)] = 0.041	Hydrogen site location: inferred from neighbouring sites
*wR*(*F*^2^) = 0.088	H-atom parameters constrained
*S* = 1.10	*w* = 1/[σ^2^(*F*_o_^2^) + (0.0387*P*)^2^ + 1.547*P*] where *P* = (*F*_o_^2^ + 2*F*_c_^2^)/3
4046 reflections	(Δ/σ)_max_ = 0.003
240 parameters	Δρ_max_ = 0.33 e Å^−^^3^
0 restraints	Δρ_min_ = −0.45 e Å^−^^3^

### Special details

**Table:**

Geometry. All e.s.d.'s (except the e.s.d. in the dihedral angle between two l.s. planes) are estimated using the full covariance matrix. The cell e.s.d.'s are taken into account individually in the estimation of e.s.d.'s in distances, angles and torsion angles; correlations between e.s.d.'s in cell parameters are only used when they are defined by crystal symmetry. An approximate (isotropic) treatment of cell e.s.d.'s is used for estimating e.s.d.'s involving l.s. planes.
Refinement. Refinement of *F*^2^ against ALL reflections. The weighted *R*-factor *wR* and goodness of fit *S* are based on *F*^2^, conventional *R*-factors *R* are based on *F*, with *F* set to zero for negative *F*^2^. The threshold expression of *F*^2^ > σ(*F*^2^) is used only for calculating *R*-factors(gt) *etc*. and is not relevant to the choice of reflections for refinement. *R*-factors based on *F*^2^ are statistically about twice as large as those based on *F*, and *R*- factors based on ALL data will be even larger.

### Fractional atomic coordinates and isotropic or equivalent isotropic displacement parameters (Å^2^)

**Table:**

	*x*	*y*	*z*	*U*_iso_*/*U*_eq_	
Cu1	0.76191 (2)	0.23448 (3)	0.685956 (9)	0.02244 (10)	
N1	0.90110 (15)	0.1958 (2)	0.65733 (6)	0.0239 (4)	
N2	1.05863 (17)	0.0889 (2)	0.64129 (7)	0.0300 (5)	
N3	1.04251 (15)	0.2230 (2)	0.61944 (6)	0.0220 (4)	
N4	1.10175 (16)	0.2341 (2)	0.54782 (6)	0.0247 (4)	
N5	1.02130 (17)	0.3031 (3)	0.52517 (7)	0.0332 (5)	
N6	1.01910 (18)	0.2435 (3)	0.48737 (7)	0.0349 (5)	
O1	0.70071 (13)	0.31913 (19)	0.63234 (5)	0.0274 (4)	
H1W	0.6484	0.2655	0.6231	0.033*	
H2W	0.6787	0.4113	0.6352	0.033*	
O2	0.82932 (13)	0.1504 (2)	0.73862 (5)	0.0296 (4)	
H3W	0.8809	0.2075	0.7469	0.036*	
H4W	0.7954	0.1060	0.7583	0.036*	
O3	0.69989 (14)	−0.0117 (2)	0.66866 (6)	0.0377 (5)	
H5W	0.7299	−0.0835	0.6829	0.045*	
H6W	0.6351	−0.0260	0.6755	0.045*	
O4	0.62735 (13)	0.28272 (19)	0.71502 (5)	0.0262 (4)	
H7W	0.5760	0.2261	0.7063	0.031*	
H8W	0.6359	0.2859	0.7418	0.031*	
O5	0.79519 (13)	0.50995 (19)	0.70084 (5)	0.0294 (4)	
O6	0.97927 (13)	0.5740 (2)	0.68985 (5)	0.0306 (4)	
O7	0.84885 (15)	0.6278 (2)	0.63497 (5)	0.0325 (4)	
O8	0.84941 (15)	0.77569 (18)	0.69968 (5)	0.0304 (4)	
O9	0.54758 (15)	0.1536 (2)	0.59423 (5)	0.0363 (4)	
H9W	0.5344	0.1744	0.5683	0.044*	
H10W	0.4907	0.1616	0.6085	0.044*	
O10	0.5000	0.8423 (3)	0.7500	0.0351 (6)	
H11W	0.4932	0.9044	0.7293	0.042*	
C1	0.94835 (18)	0.2833 (3)	0.62931 (7)	0.0237 (5)	
H1A	0.9201	0.3741	0.6180	0.028*	
C2	0.9720 (2)	0.0775 (3)	0.66390 (8)	0.0304 (6)	
H2A	0.9598	−0.0033	0.6826	0.036*	
C3	1.1218 (2)	0.2839 (3)	0.59085 (7)	0.0271 (5)	
H3A	1.1207	0.3958	0.5920	0.033*	
H3B	1.1924	0.2496	0.6000	0.033*	
C4	1.15259 (19)	0.1279 (3)	0.52359 (7)	0.0245 (5)	
C5	1.0988 (2)	0.1353 (3)	0.48467 (8)	0.0280 (5)	
C6	1.1307 (2)	0.0434 (3)	0.45077 (8)	0.0380 (7)	
H6A	1.0950	0.0472	0.4247	0.046*	
C7	1.2158 (2)	−0.0516 (3)	0.45735 (10)	0.0442 (7)	
H7A	1.2385	−0.1144	0.4353	0.053*	
C8	1.2701 (2)	−0.0572 (3)	0.49644 (10)	0.0433 (7)	
H8A	1.3287	−0.1227	0.4995	0.052*	
C9	1.2399 (2)	0.0309 (3)	0.53082 (9)	0.0340 (6)	
H9A	1.2755	0.0258	0.5569	0.041*	
S1	0.86691 (4)	0.62224 (6)	0.680761 (18)	0.02084 (14)	

### Atomic displacement parameters (Å^2^)

**Table:**

	*U*^11^	*U*^22^	*U*^33^	*U*^12^	*U*^13^	*U*^23^
Cu1	0.01695 (15)	0.03034 (19)	0.02010 (17)	−0.00004 (11)	0.00198 (11)	0.00009 (11)
N1	0.0208 (10)	0.0252 (11)	0.0259 (11)	0.0019 (8)	0.0041 (8)	0.0001 (8)
N2	0.0281 (11)	0.0278 (11)	0.0344 (13)	0.0062 (9)	0.0069 (9)	0.0071 (9)
N3	0.0216 (10)	0.0235 (10)	0.0208 (10)	0.0011 (8)	0.0032 (8)	0.0011 (8)
N4	0.0216 (10)	0.0311 (11)	0.0215 (11)	0.0046 (8)	0.0014 (8)	0.0020 (8)
N5	0.0289 (12)	0.0416 (13)	0.0292 (12)	0.0109 (10)	0.0005 (9)	0.0034 (10)
N6	0.0296 (12)	0.0479 (14)	0.0270 (12)	0.0077 (10)	−0.0029 (9)	0.0014 (10)
O1	0.0290 (9)	0.0262 (9)	0.0270 (9)	0.0039 (7)	−0.0024 (7)	−0.0009 (7)
O2	0.0226 (9)	0.0409 (11)	0.0252 (9)	−0.0061 (7)	0.0014 (7)	0.0041 (8)
O3	0.0296 (10)	0.0339 (11)	0.0494 (12)	−0.0008 (8)	−0.0039 (9)	−0.0045 (9)
O4	0.0200 (8)	0.0334 (9)	0.0252 (9)	−0.0042 (7)	0.0025 (7)	−0.0016 (7)
O5	0.0286 (9)	0.0254 (9)	0.0345 (10)	−0.0078 (7)	0.0075 (8)	−0.0030 (7)
O6	0.0190 (8)	0.0371 (10)	0.0355 (11)	−0.0002 (7)	−0.0033 (7)	0.0005 (8)
O7	0.0344 (10)	0.0431 (11)	0.0197 (9)	0.0051 (8)	−0.0020 (7)	0.0007 (8)
O8	0.0414 (11)	0.0213 (9)	0.0285 (10)	−0.0001 (7)	0.0031 (8)	−0.0014 (7)
O9	0.0346 (10)	0.0515 (12)	0.0228 (10)	0.0007 (9)	−0.0018 (8)	0.0052 (8)
O10	0.0373 (15)	0.0287 (14)	0.0387 (16)	0.000	−0.0132 (12)	0.000
C1	0.0227 (12)	0.0253 (12)	0.0230 (12)	0.0054 (9)	0.0028 (9)	0.0014 (10)
C2	0.0293 (13)	0.0261 (13)	0.0362 (15)	0.0037 (10)	0.0091 (11)	0.0080 (11)
C3	0.0259 (12)	0.0323 (14)	0.0234 (13)	−0.0025 (10)	0.0042 (10)	0.0004 (10)
C4	0.0232 (12)	0.0266 (13)	0.0240 (13)	0.0002 (9)	0.0045 (9)	0.0018 (10)
C5	0.0265 (13)	0.0324 (14)	0.0251 (14)	−0.0007 (10)	0.0010 (10)	0.0025 (10)
C6	0.0394 (16)	0.0489 (17)	0.0257 (15)	−0.0065 (13)	0.0023 (12)	−0.0080 (12)
C7	0.0467 (18)	0.0411 (17)	0.0453 (19)	−0.0024 (14)	0.0146 (14)	−0.0121 (14)
C8	0.0323 (16)	0.0386 (17)	0.059 (2)	0.0095 (12)	0.0086 (14)	−0.0033 (14)
C9	0.0267 (13)	0.0375 (15)	0.0376 (16)	0.0063 (11)	−0.0033 (11)	0.0043 (12)
S1	0.0193 (3)	0.0228 (3)	0.0205 (3)	−0.0010 (2)	0.0009 (2)	−0.0003 (2)

### Geometric parameters (Å, °)

**Table:**

Cu1—O4	1.974 (2)	O4—H8W	0.8505
Cu1—O1	1.985 (2)	O5—S1	1.473 (2)
Cu1—O2	1.988 (2)	O6—S1	1.488 (2)
Cu1—N1	2.002 (2)	O7—S1	1.459 (2)
Cu1—O3	2.331 (2)	O8—S1	1.475 (2)
N1—C1	1.312 (3)	O9—H9W	0.8501
N1—C2	1.368 (3)	O9—H10W	0.8499
N2—C2	1.310 (3)	O10—H11W	0.8500
N2—N3	1.363 (3)	C1—H1A	0.9300
N3—C1	1.329 (3)	C2—H2A	0.9300
N3—C3	1.451 (3)	C3—H3A	0.9700
N4—C4	1.360 (3)	C3—H3B	0.9700
N4—N5	1.362 (3)	C4—C5	1.391 (3)
N4—C3	1.442 (3)	C4—C9	1.393 (3)
N5—N6	1.299 (3)	C5—C6	1.397 (3)
N6—C5	1.372 (3)	C6—C7	1.358 (4)
O1—H1W	0.8501	C6—H6A	0.9300
O1—H2W	0.8499	C7—C8	1.398 (4)
O2—H3W	0.8501	C7—H7A	0.9300
O2—H4W	0.8499	C8—C9	1.384 (4)
O3—H5W	0.8499	C8—H8A	0.9300
O3—H6W	0.8500	C9—H9A	0.9300
O4—H7W	0.8499		
			
O4—Cu1—O1	89.92 (7)	N1—C1—H1A	125.0
O4—Cu1—O2	92.39 (7)	N3—C1—H1A	125.0
O1—Cu1—O2	177.58 (7)	N2—C2—N1	113.6 (2)
O4—Cu1—N1	177.18 (7)	N2—C2—H2A	123.2
O1—Cu1—N1	90.16 (8)	N1—C2—H2A	123.2
O2—Cu1—N1	87.50 (8)	N4—C3—N3	111.5 (2)
O4—Cu1—O3	91.14 (7)	N4—C3—H3A	109.3
O1—Cu1—O3	90.96 (7)	N3—C3—H3A	109.3
O2—Cu1—O3	89.72 (7)	N4—C3—H3B	109.3
N1—Cu1—O3	91.67 (7)	N3—C3—H3B	109.3
C1—N1—C2	103.7 (2)	H3A—C3—H3B	108.0
C1—N1—Cu1	127.68 (16)	N4—C4—C5	104.0 (2)
C2—N1—Cu1	128.52 (16)	N4—C4—C9	133.5 (2)
C2—N2—N3	102.88 (19)	C5—C4—C9	122.5 (2)
C1—N3—N2	109.89 (19)	N6—C5—C4	108.5 (2)
C1—N3—C3	128.3 (2)	N6—C5—C6	130.8 (2)
N2—N3—C3	121.84 (19)	C4—C5—C6	120.6 (2)
C4—N4—N5	110.47 (19)	C7—C6—C5	117.5 (3)
C4—N4—C3	131.0 (2)	C7—C6—H6A	121.3
N5—N4—C3	118.5 (2)	C5—C6—H6A	121.3
N6—N5—N4	108.1 (2)	C6—C7—C8	121.5 (3)
N5—N6—C5	108.9 (2)	C6—C7—H7A	119.2
Cu1—O1—H1W	112.0	C8—C7—H7A	119.2
Cu1—O1—H2W	112.2	C9—C8—C7	122.5 (3)
H1W—O1—H2W	107.5	C9—C8—H8A	118.8
Cu1—O2—H3W	110.6	C7—C8—H8A	118.8
Cu1—O2—H4W	124.5	C8—C9—C4	115.3 (3)
H3W—O2—H4W	115.1	C8—C9—H9A	122.3
Cu1—O3—H5W	113.8	C4—C9—H9A	122.3
Cu1—O3—H6W	112.8	O7—S1—O5	111.25 (11)
H5W—O3—H6W	100.0	O7—S1—O8	110.46 (10)
Cu1—O4—H7W	111.9	O5—S1—O8	109.00 (10)
Cu1—O4—H8W	112.0	O7—S1—O6	109.24 (11)
H7W—O4—H8W	114.9	O5—S1—O6	108.10 (11)
H9W—O9—H10W	109.9	O8—S1—O6	108.73 (11)
N1—C1—N3	109.9 (2)		
			
O4—Cu1—N1—C1	−53.0 (16)	N5—N4—C3—N3	76.2 (3)
O1—Cu1—N1—C1	38.7 (2)	C1—N3—C3—N4	−87.7 (3)
O2—Cu1—N1—C1	−140.7 (2)	N2—N3—C3—N4	93.6 (3)
O3—Cu1—N1—C1	129.7 (2)	N5—N4—C4—C5	−0.1 (3)
O4—Cu1—N1—C2	123.1 (14)	C3—N4—C4—C5	−178.0 (2)
O1—Cu1—N1—C2	−145.3 (2)	N5—N4—C4—C9	177.9 (3)
O2—Cu1—N1—C2	35.3 (2)	C3—N4—C4—C9	−0.1 (5)
O3—Cu1—N1—C2	−54.3 (2)	N5—N6—C5—C4	0.4 (3)
C2—N2—N3—C1	−0.8 (3)	N5—N6—C5—C6	−178.1 (3)
C2—N2—N3—C3	178.1 (2)	N4—C4—C5—N6	−0.2 (3)
C4—N4—N5—N6	0.3 (3)	C9—C4—C5—N6	−178.4 (2)
C3—N4—N5—N6	178.5 (2)	N4—C4—C5—C6	178.5 (2)
N4—N5—N6—C5	−0.4 (3)	C9—C4—C5—C6	0.3 (4)
C2—N1—C1—N3	−0.3 (3)	N6—C5—C6—C7	178.1 (3)
Cu1—N1—C1—N3	176.54 (15)	C4—C5—C6—C7	−0.3 (4)
N2—N3—C1—N1	0.7 (3)	C5—C6—C7—C8	−0.3 (4)
C3—N3—C1—N1	−178.1 (2)	C6—C7—C8—C9	1.1 (5)
N3—N2—C2—N1	0.7 (3)	C7—C8—C9—C4	−1.0 (4)
C1—N1—C2—N2	−0.3 (3)	N4—C4—C9—C8	−177.2 (3)
Cu1—N1—C2—N2	−177.05 (17)	C5—C4—C9—C8	0.4 (4)
C4—N4—C3—N3	−106.0 (3)		

### Hydrogen-bond geometry (Å, °)

**Table:**

*D*—H···*A*	*D*—H	H···*A*	*D*···*A*	*D*—H···*A*
O1—H1W···O9	0.85	1.82	2.662 (3)	170
O2—H3W···O10^i^	0.85	1.89	2.723 (2)	165
O9—H9W···N6^ii^	0.85	2.00	2.836 (3)	168
O10—H11W···O6^iii^	0.85	1.93	2.771 (2)	170
O10—H11W···S1^iii^	0.85	2.88	3.6458 (18)	150
O1—H2W···N2^iii^	0.85	2.16	2.951 (3)	155
O3—H5W···O8^iv^	0.85	1.99	2.789 (3)	156
O4—H7W···O6^v^	0.85	1.86	2.697 (2)	170
O4—H7W···S1^v^	0.85	2.87	3.6842 (19)	162
O9—H10W···O7^v^	0.85	1.99	2.824 (3)	165
O9—H10W···S1^v^	0.85	2.80	3.582 (2)	154
O3—H6W···O6^v^	0.85	2.19	2.943 (3)	148
O2—H4W···O5^vi^	0.85	1.92	2.766 (2)	174
O2—H4W···S1^vi^	0.85	2.82	3.571 (2)	148
O4—H8W···O8^vi^	0.85	1.85	2.702 (2)	175
O4—H8W···S1^vi^	0.85	2.82	3.5687 (18)	147

Symmetry codes: (i) *x*+1/2, *y*−1/2, *z*; (ii) −*x*+3/2, −*y*+1/2, −*z*+1; (iii) *x*−1/2, *y*+1/2, *z*; (iv) *x*, *y*−1, *z*; (v) *x*−1/2, *y*−1/2, *z*; (vi) −*x*+3/2, *y*−1/2, −*z*+3/2.
